# Alterations in the Interplay between Neurons, Astrocytes and Microglia in the Rat Dentate Gyrus in Experimental Models of Neurodegeneration

**DOI:** 10.3389/fnagi.2017.00296

**Published:** 2017-09-11

**Authors:** Daniele Lana, Filippo Ugolini, Daniele Nosi, Gary L. Wenk, Maria G. Giovannini

**Affiliations:** ^1^Department of Health Sciences, Section of Pharmacology and Clinical Oncology, University of Florence Florence, Italy; ^2^Department of Experimental and Clinical Medicine, University of Florence Florence, Italy; ^3^Department of Psychology, The Ohio State University Columbus, OH, United States

**Keywords:** inflammaging, confocal microscopy, apoptosis, phagocytosis, phagoptosis, CX3CL1, MAP2, S100

## Abstract

The hippocampus is negatively affected by aging and neurodegenerative diseases leading to impaired learning and memory abilities. A diverse series of progressive modifications in the intercellular communication among neurons, astrocytes and microglia occur in the hippocampus during aging or inflammation. A detailed understanding of the neurobiological modifications that contribute to hippocampal dysfunction may reveal new targets for therapeutic intervention. The current study focussed on the interplay between neurons and astroglia in the Granule Layer (GL) and the Polymorphic Layer (PL) of the Dentate Gyrus (DG) of adult, aged and LPS-treated rats. In GL and PL of aged and LPS-treated rats, astrocytes were less numerous than in adult rats. In GL of LPS-treated rats, astrocytes acquired morphological features of reactive astrocytes, such as longer branches than was observed in adult rats. Total and activated microglia increased in the aged and LPS-treated rats, as compared to adult rats. In the GL of aged and LPS-treated rats many neurons were apoptotic. Neurons decreased significantly in GL and PL of aged but not in rats treated with LPS. In PL of aged and LPS-treated rats many damaged neurons were embraced by microglia cells and were infiltrated by branches of astrocyte, which appeared to be bisecting the cell body, forming triads. Reactive microglia had a scavenging activity of dying neurons, as shown by the presence of neuronal debris within their cytoplasm. The levels of the chemokine fractalkine (CX3CL1) increased in hippocampal homogenates of aged rats and rats treated with LPS, and CX3CL1 immunoreactivity colocalized with activated microglia cells. Here we demonstrated that in the DG of aged and LPS-treated rats, astrocytes and microglia cooperate and participate in phagocytosis/phagoptosis of apoptotic granular neurons. The differential expression/activation of astroglia and the alteration of their intercommunication may be responsible for the different susceptibility of the DG in comparison to the CA1 and CA3 hippocampal areas to neurodegeneration during aging and inflammation.

## Introduction

Inflammaging is a chronic, low-grade upregulation of pro-inflammatory mechanisms that may be a prodrome of Alzheimer’s Disease (AD; Franceschi et al., 2007; Giunta et al., 2008; Salminen et al., 2012; Baylis et al., 2013; Salvioli et al., 2013; Deleidi et al., 2015). Aging is considered a primary risk factor for dementia of the Alzheimer’s type and neuroinflammation is likely an important component of AD and other brain disorders such as depression, Parkinson’s disease (PD) and traumatic brain injury (TBI; Stoll et al., 2000; Vitkovic et al., 2000; Morganti-Kossmann et al., 2001; Strle et al., 2001; Orr et al., 2002; Williamson and Bilbo, 2013; Szot et al., 2017).

However, the association between aging, inflammation and neurodegeneration in the progression of these disorders is still unclear. The neuroinflammatory response is a complex cascade of molecular and cellular changes which involve different cells, multiple proteins and a complex time-course that is only starting to be understood. Elevated levels of proinflammatory molecules augment the deposition of β-amyloid within neurons (Blasko et al., 1999; Sastre et al., 2003; Giunta et al., 2008; Mercatelli et al., 2016). We have previously shown that inflammaging can modify the neuron-astrocyte-microglia interactions in CA1 and CA3 (Cerbai et al., 2012; Lana et al., 2014, 2016, 2017). We hypothesized that this mechanism may be important not only for normal brain aging, but also for AD (Mercatelli et al., 2016).

The dentate gyrus (DG) has striking anatomical differences (Amaral et al., 2007) and distinct functions in comparison to CA1 and CA3, and contributes to specific kinds of information processing (Kesner, 2013). Indeed, the DG is the first link in the canonical hippocampal trisynaptic circuit, receiving major inputs from the entorhinal cortex (EC) and sending its outputs via the mossy fibers (Amaral, 1993; Amaral and Lavenex, 2007; Witter, 2007) that form synapses at proximal apical dendrites of CA3 pyramidal cells.

Clinically, aging and brain traumatic injuries lead to declining hippocampal functions and memory impairment in almost half of the Western population over 60 years of age (Small et al., 2002; Hedden and Gabrieli, 2004). Intercellular communication is raising particular interest not only because it can change during aging but also because it can provide insights into the aging process itself. Therefore, with the aging of Western population, understanding the neurobiological changes that may lead to dysfunction of the hippocampus and memory impairment may help to identify new targets for therapeutic interventions that may maintain hippocampal function. It is not clear if the cellular response mechanisms to the same insult are similar or different in the different areas of the hippocampus, CA1, CA3 and DG, and how the cell respond to it. Using models of aging and inflammation, we had previously demonstrated that while astrocytes decrease and show signs of clasmatodendrosis both in CA1 and CA3 of aged but not of LPS-treated rats, microglia behave differently in the two regions, decreasing in CA1 and increasing in CA3 of aged rats (Cerbai et al., 2012; Lana et al., 2014; Mercatelli et al., 2016). Therefore, guided by our previous discoveries in areas CA1 and CA3 of the hippocampus (Cerbai et al., 2012; Lana et al., 2014, 2017), the aim of the present research was to investigate in the DG whether and how the interaction between glial cells and neurons change in rat models of normal brain aging and during an acute inflammatory insult. In the light of our previous investigations in this field, the different response of DG in the same animal models may be of interest to understand the differential responsivity of this area to stress stimuli.

## Experimental Procedures

### Animals

Male adult Wistar rats were used (3 and 22 months old, Harlan, Milano, Italy). Rats were housed in cages with food and water* ad libitum*, in a temperature controlled room (23 ± 1°C, 12 h light–12 h dark cycle). Experiments were authorized by the IACUC of the University of Florence and by the Italian Ministry of Health (Italian Law on Animal Welfare, DL 116/92). According to the law, we did all efforts to fulfill the 3Rs requirements. The total number of rats used was: adult rats, *n* = 6; aged rats, *n* = 6; LPS-treated rats: *n* = 7.

### LPS Treatment

Experiments on LPS-treated rats were performed in the Department of Psychology, The Ohio State University, Columbus, OH, USA (Hauss-Wegrzyniak et al., 1998; Cerbai et al., 2012; Lana et al., 2016) in accordance with the National Institute of Health Guide for the Care and Use of Laboratory Animals (NIH Publications No. 80-23) revised 1996; formal approval to conduct the experiments was obtained from the Institutional Animal Care and Use Committee (approval number 2008A0028). Male rats (3 months) old were used. Briefly, LPS or artificial cerebrospinal fluid (aCSF, in mM: 140 NaCl; 3.0 KCl; 2.5 CaCl_2_; 1.0 MgCl_2_; 1.2 Na_2_HPO_4_, pH 7.4) was administered for 4 weeks to adult rats using an Alzet osmotic minipump containing 1.6 μg/ml LPS (Sigma; *E. coli*, serotype 055:B5, TCA extraction). The minipump was attached to a chronic indwelling cannula (Model 3280P, osmotic pump connect, 28 gauge, Plastics One, Inc., Roanoke, VA, USA) that was positioned stereotaxically into the 4th ventricle (coordinates on the midline: −2.5 mm posterior to Lambda, 7 mm ventral to the dura). The animal was deeply anesthetized with isoflurane for the duration of surgery. Post-operative care included a local antibiotic applied to the exposed skull and scalp (1% chloramphenicol), a long-acting topical anesthetic applied locally to the scalp (Bupivacaine), and 4 ml of sterile isotonic saline injected s.c. to prevent dehydration. During recovery, body weight and general behavior were monitored and at the end of the 4 weeks of LPS administration, rats were anesthetized and perfused with paraformaldehyde (see below) to collect the brain for immunohistochemical analyses.

### Fluorescent Immunohistochemistry

Rats, deeply anesthetized with Zoletil, were perfused transcardially with ice-cold paraformaldehyde (500 ml of 4% solution in phosphate-buffered saline (PBS) pH 7.4). The brains were collected, postfixed for 4 h in 4% paraformaldehyde and then cryoprotected for 48–72 h in 18% sucrose/PBS solution. Coronal sections (40 μm) were cut with a cryostat, placed in 1 ml of anti-freeze solution and stored at −20°C until use (Cerbai et al., 2012; Lana et al., 2016). Immunostaining was performed on coronal sections with the free-floating method (Giovannini, 2002; Lana et al., 2016).

The primary and secondary antibodies used for the immunohistochemical and Western Blot analyses are shown in Table 1.

**Table 1:** Antibodies used for immunohistochemistry and Western Blot.

Target	Antigen	Supplier	Catalog #	Antibody	Host	Usage	Conc
**Immunohistochemistry**					
Neurons	NeuN	Millipore	MAB377	Monoclonal	Ms	Primary	1:200
Neurons	NeuN	Millipore	MAB377X	Monoclonal conj	Ms	Primary	1:200
Neurons	MAP2	Chemicon	AB5622	Polyclonal	Rb	Primary	1:300
Astrocytes	GFAP	Dako	Z0334	Policlonal	Rb	Primary	1:500
Astrocytes (triple labeling IHC)	GFAP	Millipore	MAB3402X	Monoclonal	Ms	Primary	1:500
Astrocytes	S100 beta	Abcam	14849	Monoclonal	Ms	Primary	1:300
Total microglia	Iba1	Wako	016-20001	Policlonal	Rb	Primary	1:300
Activated microglia	OX6	BD	554926	Monoclonal	Ms	Primary	1:200
Cytocrome C	CytC	BD	556432	Monoclonal	Ms	Primary	1:200
CX3CL1	CX3CL1	Abcam	AB-25088	Polyclonal	Rb	Primary	1:400
Rabbit FC	Rabbit FC	Life technologies	A21206	Polyclonal	Dn	Secondary Alexa Fluor 488	1:400
Mouse FC (triple labeling IHC)	Mouse FC	Life technologies	A31570	Polyclonal	Dn	Secondary Alexa Fluor 555	1:400
Rabbit FC	Rabbit FC	Life technologies	A31577	Polyclonal	Gt	Secondary Alexa Fluor 635	1:400
**Western Blot**					
CX3CL1	CX3CL1	Abcam	AB25088	Polyclonal	Rb	Primary	1:300
Actin	Actin	Sigma	A-2066	Polyclonal	Rb	Primary	1:10000

Colocalization of different antigens in the same cell was carried out using different primary and secondary antibodies with double or triple-labeling laser confocal microscopy and digital subslicing (see below).

#### Day 1: Primary Antibodies

Selected brain sections containing the dorsal hippocampi were placed in multiwells with 1 ml of PBS-TX. Sections were rinsed three times for 5 min with 500 μl PBS-TX under slight agitation at room temperature (RT), blocked with 500 μl BB (10% Normal Goat Serum, 10% Normal Horse Serum, 0.05% NaN_3_ in PBS-TX) for 1 h under agitation at RT, and washed three times as above. For single immunostaining, sections were incubated overnight (O/N) at 4°C under slight agitation with the primary antibody, dissolved in 250 μl of BB at the appropriate dilution (see Table 1). For double immunostaining, sections were incubated O/N with a solution containing two or three primary antibodies, respectively, diluted in 250 μl of BB at the appropriate concentrations (see Table 1).

#### Day 2: Secondary Antibodies

Sections were washed three times as above and incubated for 2 h in the dark at RT under slight agitation with the appropriate secondary antibody diluted in 250 μl of BB. For double immunostaining, sections were incubated for 2 h in the dark at RT under slight agitation with a solution containing the appropriate fluorescent secondary antibodies diluted in 250 μl of BB. Sections were then washed as above. For triple immunostaining the sections were incubated for 2 h in the dark at RT under agitation with a fluorophore-conjugated primary antibody, diluted in 250 μl of BB (Table 1). Sections were thoroughly washed with PBS-TX and then with 1 ml of distilled H_2_O in the dark, mounted on gelatinized microscopy slides, left to dry and covered in the dark with coverslips with a mounting medium (Vectashield, Hard set mounting medium with DAPI, Vector Laboratories, Burlingame, CA, USA) containing DAPI to counterstain nuclei. Slides were kept in the fridge until microscopy analysis.

#### Day 3: Qualitative and Quantitative Analyses

Confocal scans were taken at 0.3 μm z-step, keeping constant all the parameters (pinhole, contrast and brightness), using a LEICA TCS SP8 confocal laser scanning microscope (Leica Microsystems CMS GmbH, Mannheim, Germany). Voxel size was 6.75 × 10^−3^ μm^3^. Images were converted to green, red or blue using ImageJ (National Institute of Health). Qualitative analyses were performed on 3D renderings obtained using ImageJ 3D viewer from the stacks of confocal scans. All the counts, measures and image analysis were performed blind by two researchers with the ImageJ program (freeware provided by National Institute of Health^1^) and the results were averaged.

**Figure 1:**
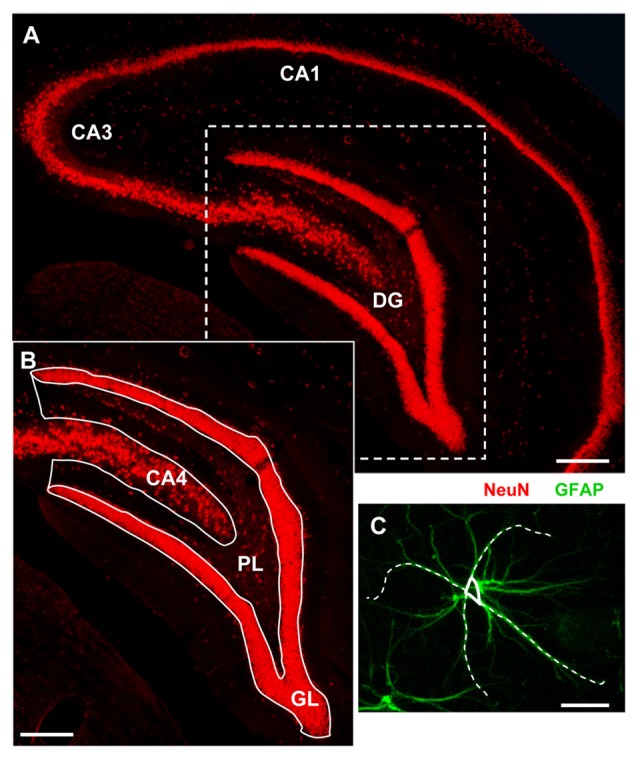
Representative image of the region of interest (ROI) for the analyses. **(A)** Fluorescent immunostaining of neurons with anti-NeuN antibody in the dorsal hippocampus of a young rat. Scale bar: 250 μm. **(B)** Magnification of the framed area in **(A)** schematically showing the dentate gyrus (DG) subregions: Granular Layer (GL) and Polymorphic Layer (PL). Hippocampal CA4 is also shown. Scale bar: 100 μm. **(C)** Schematic diagram showing the method used to measure the length of principal GFAP+ astrocytes branches. Scale bar: 10 μm.

For quantitative analysis images were acquired at 20× magnification with an Olympus BX63 microscope equipped with an Olympus DP 50 digital camera (Olympus, Milan, Italy). Analyses were carried out blind in the DG of the hippocampus DG (see Figure 1A Region of Interest, ROI; Lorente de Nó, 1934; Li et al., 1994) using ImageJ. Quantitative analyses were carried out separately in the following subregions *Granular Layer* (GL) and *Polymorphic Layer* (PL; Amaral and Lavenex, 2007, see Figure 1B). All quantifications were done independently by two researchers, and results were averaged. Three coronal sections (spaced by 150 μm, starting at about −2.8 mm from bregma) containing the DG were analyzed.

Quantitative analyses of NeuN^+^ neurons, GFAP^+^ astrocytes, IBA1^+^ total microglia, OX6^+^ activated microglia, CytC^+^ apoptotic neurons, neuron-astrocyte-microglia triads, were performed separately in GL and PL of the DG. All counts were performed blind by two experimenters and results were averaged. Digitized images, acquired keeping all the parameters (contrast and brightness) constant using a 10× objective, were transformed into TIFF files and thresholded using ImageJ. Care was taken to maintain the same threshold in all sections from the same experiment. The area above the set threshold was calculated in pixels. Areas of GL and PL were calculated in mm^2^ and the counts of immunopositive cells, or triads were expressed as number/mm^2^. Quantitation of DG granular neurons was obtained counting the number of NeuN or MAP2 positive cells in GL. The length of principal astrocyte branches was measured choosing randomly four principal branches of three GFAP+ astrocytes per ROI and results were averaged. A “triad” was defined as a neuron in direct contact with astrocyte branches of surrounding astrocyte(s) and with a microglia cell (Cerbai et al., 2012; Lana et al., 2016). The reciprocal interplay of the neurons, astrocytes and microglia in the triads was highlighted digitally sub-slicing the triad as previously reported (Cerbai et al., 2012). A 3D rendering of the sub-slice was obtained using ImageJ 3D viewer. Control immunostaining was performed omitting the primary or secondary antibodies to verify the specificity of the immunostaining.

### Western Blot

Western blot analysis of CX3CL1 was performed as previously described (Cerbai et al., 2012). Hippocampal slices (400 μm-thick), cut using a tissue chopper, were placed in an Eppendorff tube with 100 μl of ice-cold lysis buffer, and were homogenized on ice using a homogenizer directly in the Eppendorf tube (15 strokes, 1 stroke per second, on ice). Composition of the lysis buffer (in mM, unless otherwise indicated): 50 Tris-HCl, pH 7.5, 50 NaCl, 10 EGTA, 5 EDTA, 2 sodium pyrophosphate, 4 para-nitrophenylphosphate, 1 sodium orthovanadate, 1 phenylmethylsulfonyl fluoride (PMSF), 25 sodium fluoride, 2 DTT, 1 μM okadaic acid, 1 μM microcystin L-R, 20 μg/ml leupeptin, and 4 μg/ml aprotinin. After homogenization an additional 2.5 μl of PMSF was added to each tube, and protein determination was performed using Bio-Rad Protein Assay reagent (Bio-Rad, Hercules, CA, USA). An appropriate volume of 6× Loading Buffer was added to the homogenates, samples were boiled for 5 min, immediately put on ice, loaded on a 10% SDS-PAGE gel (30 μg of proteins/well) and run using standard electrophoresis. The gels were transferred electrophoretically by the iBlot dry blotting system (Invitrogen) onto 0.2 mm nitrocellulose membrane, and incubated O/N at 4°C under slight agitation with the primary antibody against CX3CL1 (Table 1) dissolved in blocking solution. The day after, the blots were incubated for 1 h with HRP-conjugated secondary antibody (1:4000 in blocking solution, Thermo Scientific, Waltham, MA, USA), were visualized with enhanced chemiluminescence (Immobilon Western, Millipore, Billerica, MA, USA), and resolved with ImageQuant 350 system (GE Healthcare, Buckinghamshire, UK). Densitometric band analysis was performed using the Image Quant TL software version 7.0 (GE Healthcare). For quantitative analysis, band density was normalized against βactin, run in the same gel.

### Statistical Analysis

Statistical analyses were performed using Graph Pad Prism (Graph Pad Software Inc., La Jolla, CA, USA). Unless otherwise stated, all statistical analyses were performed using ANOVA, followed by Newman-Keuls Multiple Comparison Test. Significativity was set at *P* < 0.05.

## Results

### Analysis of Neurons in the Dentate Gyrus of Adult, Aged and LPS-Treated Rats

**Figure 2:**
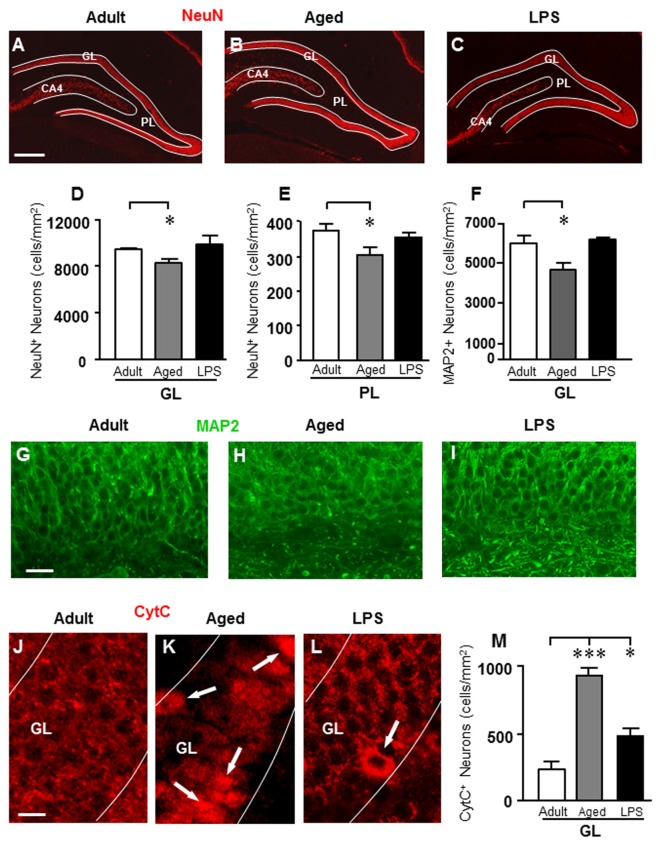
Analysis of neurons in GL and PL of adult, aged and LPS-treated rats. **(A–C)** Representative photomicrographs of NeuN immunostaining of neurons (red) in DG of an adult **(A)**, an aged **(B)** and an LPS-treated rat **(C)**. Scale bar: 200 μm. **(D,E)** Quantitative analysis of neurons/mm^2^ in DG GL **(D)** and PL **(E)** of adult (*n* = 6), aged (*n* = 5) and LPS-treated rats (*n* = 6). Neurons were significantly less numerous in GL and PL of aged rats. **(F)** Quantitative analysis of MAP2 neurons/mm^2^ in DG GL of adult (*n* = 6), aged (*n* = 5) and LPS-treated rats (*n* = 4). MAP2+ granular neurons were significantly less numerous in GL of aged rats. **(G–I)** Representative photomicrographs of MAP2 immunostaining (green) in the GL of an adult **(G)**, an aged **(H)** and an LPS-treated rat **(I)**. Scale bar: 25 μm. **(J–L)** Representative photomicrographs of CytC immunostaining (red) in the GL of an adult **(J)**, an aged **(K)** and an LPS-treated rat **(L)**. The arrows in **(K)** and **(L)** point to apoptotic neurons in GL. Scale bar: 10 μm. **(M)** Quantitative analysis of apoptotic neurons/mm^2^ in GL of adult (*n* = 4), aged (*n* = 4) and LPS-treated rats (*n* = 4). Apoptotic granular neurons were significantly more numerous in GL of aged and LPS-treated rats. Data reported in all graph bars are expressed as mean ± SEM. All statistical analyses were performed using ANOVA and Newman-Keuls Multiple Comparison Test: *P < 0.05 vs. adult rats, ***P < 0.001 vs. adult rats (see text for details).

To evaluate whether aging or LPS might cause a loss of neurons in the DG, neurons were immunostained with anti NeuN or MAP2 antibody, and counted separately in GL and PL (Figure 2).

Using the anti-NeuN antibody (Figures 2A–C), we found a significant decrease of neurons in GL and PL of aged rats in comparison to adult rats while no effect was found in LPS rats. Statistical analysis showed that in DG of aged rats, neurons decreased by 13% in GL (*P < 0.05 aged vs. adult, *F*_(2,13)_ = 3.874), and by 20% in PL (*P < 0.05 aged vs. adult, *F*_(2,12)_ = 4.212) in comparison to adult rats (Figures 2D,E).

Using the anti-MAP2 antibody (Figures 2G–I), we confirmed that granular neurons significantly decreased in GL of aged rats (−23% in comparison to adult rats; *P < 0.05 aged vs. adult, *F*_(2,12)_ = 6.483), but not in GL of LPS-treated rats (n.s., Figure 2F).

To define if the decrease of granular neurons in the DG of aged rats might be caused by apoptosis, DG sections were immonostained for CytC, one of the late markers of apoptosis (Suen et al., 2008). Figures 2J–L show the immunolabeling of CytC in GL of an adult (Figure 2J), an aged (Figure 2K) and an LPS-treated rat (Figure 2L). The arrows show neurons with increased cytoplasmic immonostaining for CytC. Quantitative analysis of CytC-positive neurons in GL is shown in Figure 2M. Statistical analysis demonstrated that CytC-positive neurons were significantly more numerous in GL of aged (+300%) and of LPS treated rats (+108%) than in adult rats (***P < 0.001 aged vs. adult; *P < 0.05 LPS vs. adult; *F*_(2,9)_ = 40.04).

### Analysis of Astrocytes in the Dentate Gyrus of Adult, Aged and LPS-Treated Rats

**Figure 3:**
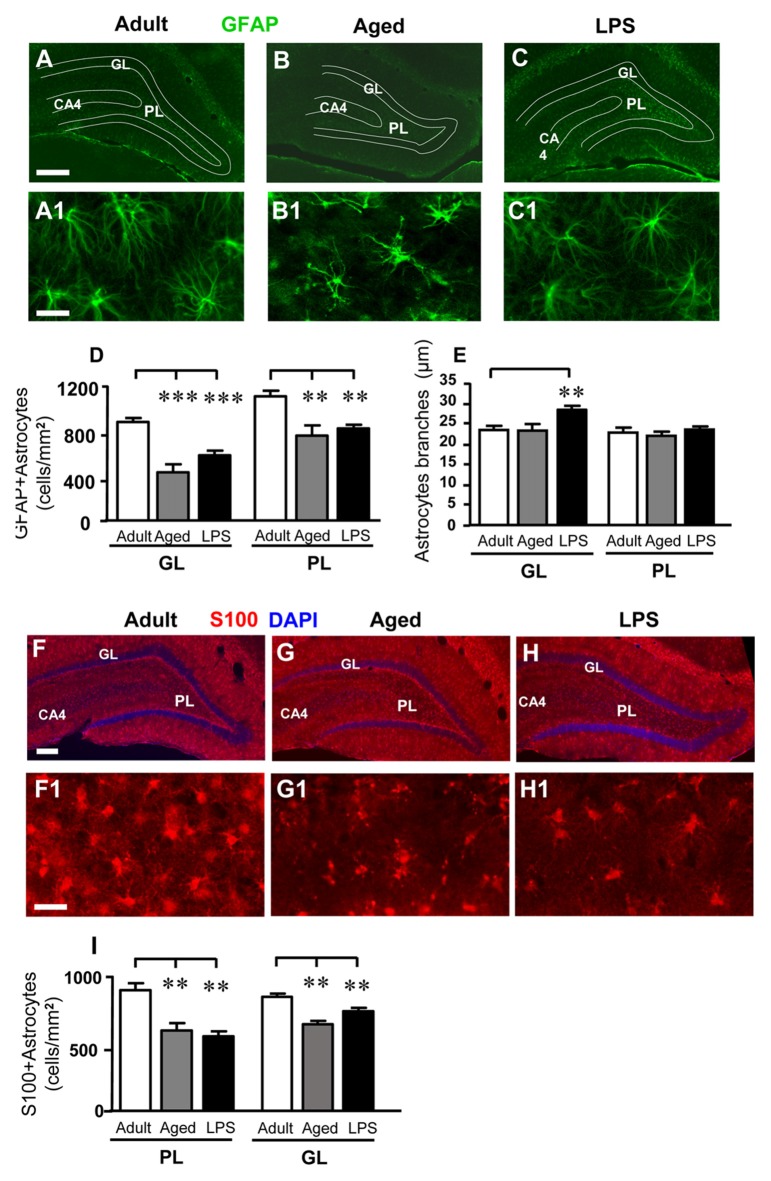
Characterization and quantitative analysis of astrocytes in GL and PL of adult, aged and LPS-treated rats.** (A–C)** Representative photomicrographs showing immunoreactivity of GFAP (green) in DG of an adult **(A)**, an aged **(B)** and an LPS-treated rat **(C)**. Scale bar: 150 μm. **(A1–C1)** Magnification of GFAP+ astrocytes in the PL of an adult **(A1)**, an aged **(B1)** and an LPS-treated rat **(C1)**. Scale bar: 25 μm.** (D)** Quantitative analysis of GFAP positive astrocytes/mm^2^ in hippocampal GL and PL of adult (*n* = 5), aged (*n* = 4) and LPS-treated rats (*n* = 6). GFAP+ astrocytes were significantly less numerous in GL and PL of aged and LPS-treated rats. **(E)** Length of principal astrocyte branches in GL and PL of adult (*n* = 5), aged (*n* = 5) and LPS-treated rats (*n* = 7). GFAP+ astrocytes branches were significantly longer in GL of LPS-treated rats. **(F–H)** Representative photomicrographs showing immunoreactivity of S100 (red) in DG of an adult **(F)**, an aged **(G)** and an LPS-treated rat **(H)**. Nuclei were counterstained with DAPI (blue). Scale bar: 100 μm. **(F1–H1)** Magnification of S100+ astrocytes in the PL of an adult **(F1)**, an aged **(G1)** and an LPS-treated rat **(H1)**. Scale bar: 50 μm. **(I)** Quantitative analysis of S100-positive astrocytes/mm^2^ in hippocampal GL and PL of adult (*n* = 6), aged (*n* = 5) and LPS-treated rats (*n* = 5). S100+ astrocytes were significantly less numerous in GL and PL of aged and LPS-treated rats. Data reported in all graph bars are expressed as mean ± SEM. All statistical analyses were performed using ANOVA and Newman-Keuls Multiple Comparison Test: **P < 0.01 vs. adult rats, ***P < 0.001 vs. adult rats (see text for details).

As shown in the representative images of Figure 3, astrocytes were immunolabeled with anti-GFAP antibody (Figures 3A–C1) and anti-S100 antibody (Figures 3F–H1), and counted separately in GP and PL of adult, aged and LPS-treated rats. Quantitative analysis of GFAP+ astrocytes, reported in Figure 3D, showed that GFAP-positive astrocytes decreased both in GL and PL of aged and LPS-treated rats. Statistical analysis demonstrated that GFAP+ astrocytes were significantly less numerous in GL of aged rats (−50%, ***P < 0.001 aged vs. adult rats, *F*_(2,16)_ = 19.67) and in GL of LPS-treated rats (−33%, **P < 0.01 LPS vs. adult rats, *F*_(2,16)_ = 10.43) in comparison to adult rats, respectively (Figure 3D). Similarly, GFAP+ astrocytes were less numerous in PL of aged rats (−31%, **P < 0.01 aged vs. adult rats, *F*_(2,16)_ = 10.43), and in PL of LPS treated rats (−25%, **P < 0.01 LPS vs. adult rats, *F*_(2,16)_ = 10.43) in comparison to adult rats, respectively (Figure 3D).

Similar results were obtained immunostaining astrocytes with a different marker, protein S100. The number of S100+ astrocytes was significantly lower in GL of aged (−33%) and of LPS-treated (−39%) rats (*P < 0.05 aged vs. adult rats, and **P < 0.01 LPs vs. adult rats; *F*_(2,13)_ = 10.04). The significant decrease of S100+ astrocytes was also evident in PL of aged (−26%) and of LPS treated (−14%) rats (***P < 0.01 aged vs. adult rats, **P < 0.01 LPS vs. adult rats, *F*_(2,13)_ = 18.28; Figure 3I).

Images of GFAP+ astrocytes taken at higher magnification in PL (Figures 3A1–C1), show that in aged rats principal branches of GFAP+ astrocytes appeared twisted and shorter, as compared to those of adult and LPS-treated rats. We thus measured the length of GFAP+ astrocytes branches (see “Materials and Methods” Section, Figure 1C) and the results are presented in Figure 3E. In the GL of LPS-treated rats, the length of astrocytes principal branches was significantly longer (+21%) than in adult rats (**P < 0.01 LPS vs. adult rats, *F*_(2,16)_ = 6.814, Figure 3E). On the contrary, in PL the length of astrocytes branches did not differ significantly among the three experimental groups (Figure 3E).

**Figure 4:**
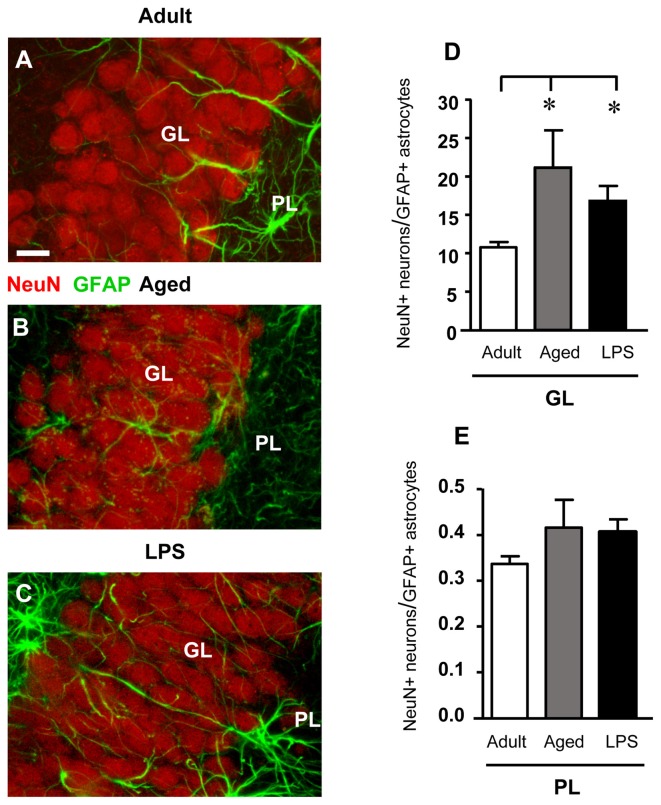
Confocal microscopy 3D renderings of double immunostaining of neurons (NeuN, red), and astrocytes (GFAP, green) in the GL of an adult **(A)**, an aged **(B)** and of an LPS-treated rat **(C)**. Scale bar: 10 μm. **(D,E)** Ratios between NeuN+ neurons and GFAP+ astrocytes in GL **(D)** and PL **(E)** of adult (*n* = 6), aged (*n* = 5) and LPS-treated (*n* = 6) rats. The ratios NeuN+ neurons/GFAP+ astrocytes increased in GL of aged and LPS-treated rats. Data reported in all graph bars are expressed as mean ± SEM. All statistical analyses were performed using ANOVA and Newman-Keuls Multiple Comparison Test: *P < 0.05 vs. adult rats (see text for details).

Figures 4A–C shows the confocal 3D rendering of astrocyte branches passing through the GL of an adult (Figure 4A), an aged (Figure 4B) and an LPS-treated rat (Figure 4C). Each image, obtained stacking 17 consecutive confocal z-scans (0.3 μm each, total thickness 5.1 μm) confirms that in LPS-treated rats astrocyte branches were significantly longer than in aged and adult rats.

We calculated the ratio between NeuN+ neurons and GFAP+ astrocytes both in GL and PL of adult, aged and LPS-treated rats to verify whether the decrease of GFAP+ astrocytes, paralleled by a decrease of neurons, might mask a possible astrocytosis. The results obtained presented in Figures 4D,E demonstrate that in GL of aged and LPS-treated rats the ratios NeuN+ neurons/GFAP+ astrocytes were significantly higher than in adult rats (+95% and +56%, respectively), and were both statistically significant (*P < 0.05 aged vs. adult rats, and LPS vs. adult rats, *F*_(2,13)_ = 4.24). In PL, the ratios NeuN+ neurons/GFAP+ astrocytes were not significantly different in the three experimental groups (*F*_(2,14)_ = 1.434, n.s.; one-way ANOVA). These data further demonstrate that no astrocytosis was present in GL and PL of aged and LPS-treated rats.

### Quantification of Total and Activated Microglia in the Dentate Gyrus of Adult, Aged and LPS-Treated Rats

**Figure 5:**
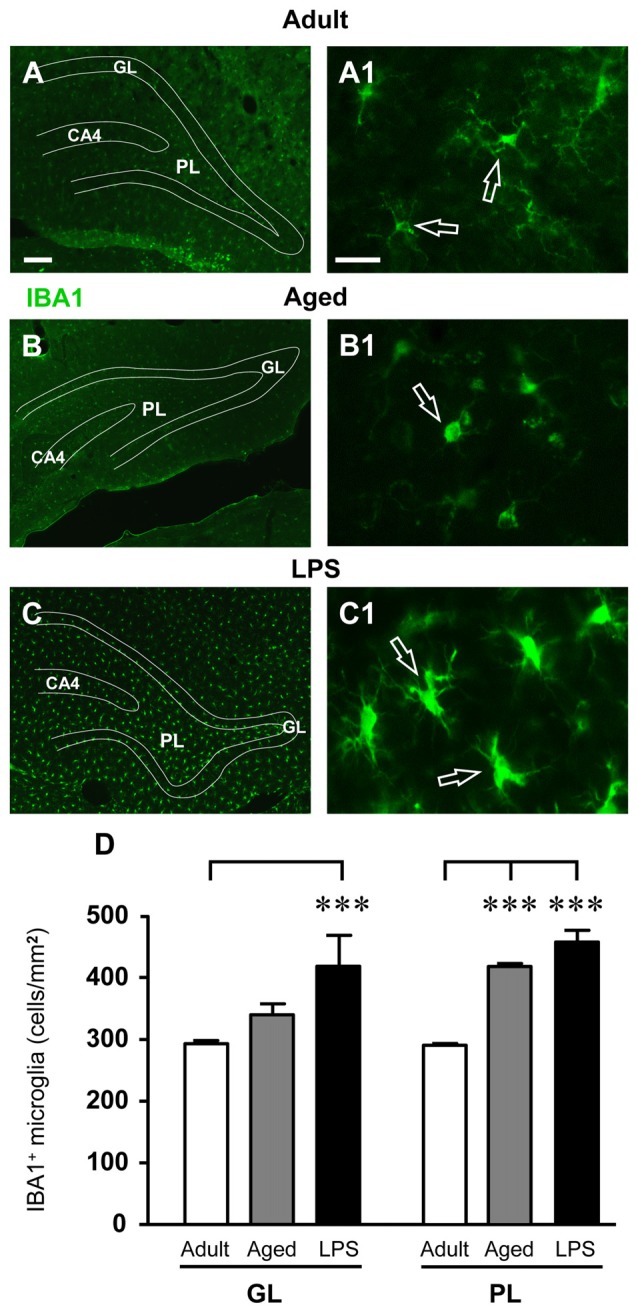
Analysis of total microglia in GL and PL of adult, aged and LPS-treated rats. **(A–C)** Representative photomicrographs of IBA1 immunostaining of total microglia (green) in DG of an adult **(A)**, an aged **(B)** and an LPS-treated rat **(C)**. Scale bar: 100 μm. **(A1–C1)** Magnification of total microglia in the PL of an adult **(A1)**, an aged **(B1)** and an LPS-treated rat **(C1)**. Scale bar: 15 μm. **(D)** Quantitative analysis of IBA1 positive microglia/mm^2^ in hippocampal GL and PL of adult (*n* = 5), aged (*n* = 5) and LPS-treated rats (*n* = 5). Microglia were significantly more numerous in GL of LPS-treated rats and in PL of aged and LPS-treated rats. Data reported in all graph bars are expressed as mean ± SEM. All statistical analyses were performed using ANOVA and Newman-Keuls Multiple Comparison Test: ***P < 0.001 vs. adult rats (see text for details).

Total microglia was identified using the fluorescent immunostaining for IBA1, as shown by the representative images of Figures 5A–C1. Quantitative analysis of IBA1-positive cells revealed that the total number of microglia significantly increased by 42% in comparison to adult rats in the GL of LPS-treated rats (***P < 0.001 LPS vs. adult rats, *F*_(2,14)_ = 4.22), while the increase found in GL of aged rats (+16% vs. adult rats) was not statistically significant. Furthermore, total microglia significantly increased both in the PL of aged (+44%, ***P < 0.001 aged vs. adult rats, *F*_(2,14)_ = 56.33) and of LPS-treated rats (+58%, ***P < 0.001 LPS vs. adult rats, *F*_(2,14)_ = 56.33), in comparison to adult rats (Figure 5D).

**Figure 6:**
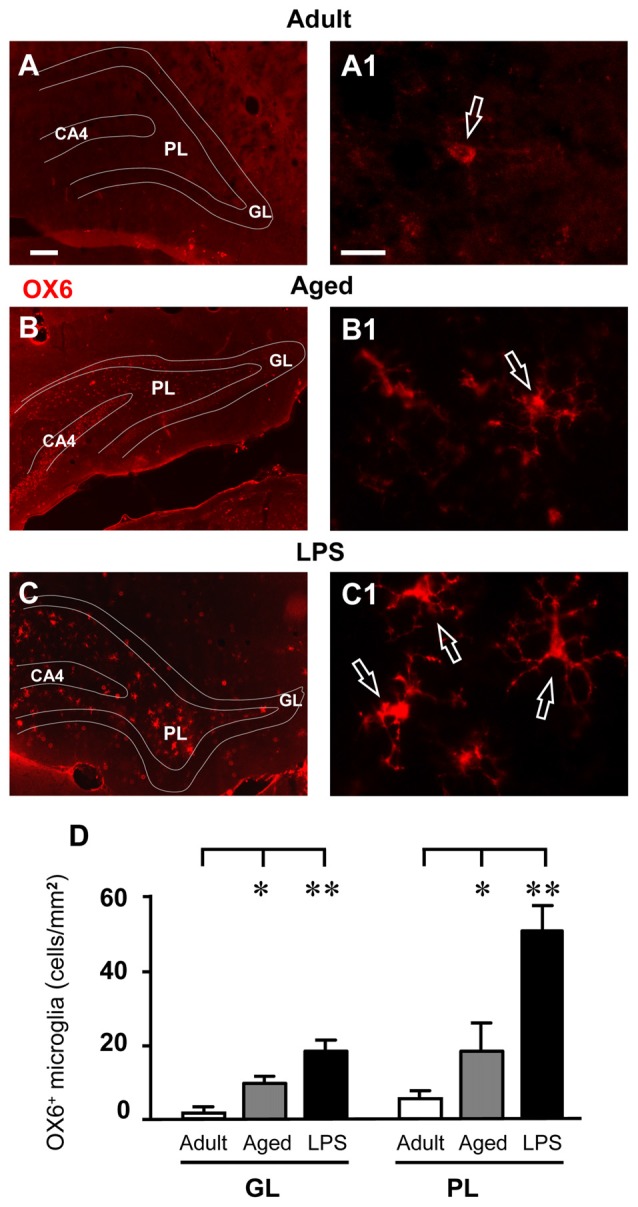
Analysis of OX6 positive, activated microglia in GL and PL of adult, aged and LPS-treated rats. **(A–C)** Representative photomicrographs of OX6 immunostaining of activated microglia (red) in DG of an adult **(A)**, an aged **(B)** and an LPS-treated rat **(C)**. Scale bar: 100 μm. **(A1–C1)** Magnification of activated microglia in PL of an adult **(A1)**, an aged **(B1)** and an LPS-treated rat **(C1)**. Scale bar: 15 μm. **(D)** Quantitative analysis of activated microglia/mm^2^ in hippocampal GL and PL of adult (*n* = 3), aged (*n* = 5) and LPS-treated rats (*n* = 4). Activated microglia cells were significantly more numerous in GL and PL of aged and LPS-treated rats. Data reported in all graph bars are expressed as mean ± SEM. All statistical analyses were performed using ANOVA and Newman-Keuls Multiple Comparison Test: *P < 0.05 vs. adult rats, **P < 0.01 vs. adult rats (see text for details).

IBA1-immunostained microglia in the DG of aged and LPS-treated rats (Figures 5B1–C1) had morphological features typical of activated microglia. Indeed, as shown in Figures 6A–C1, numerous OX6-positive, activated microglia cells were found in the GL and PL of aged and LPS-treated rats. Magnifications of OX6-positive, activated, microglia are shown in Figures 6A1–C1. Quantitative analysis demonstrated that activated microglia significantly increased both in GL and PL of aged and LPS-treated rats in comparison to adult rats (Figure 6D). Activated microglia increased by 489% in GL of aged rats (*P < 0.05 aged vs. adult rats, *F*_(2,11)_ = 11.20) and by 2160% in GL of LPS-treated rats (**P < 0.01 LPS vs. adult rats, *F*_(2,11)_ = 11.20). Activated microglia increased by 235% in PL of aged rats (*P < 0.05 aged vs. adult rats, *F*_(2,9)_ = 13.83) and by 829% in PL of LPS-treated rats (**P < 0.01 LPS vs. adult rats, *F*_(2,9)_ = 13.83), in comparison to adult rats (Figure 6D).

### Quantification of Neuron-Astrocyte-Microglia Triads in the PL of the Dentate Gyrus of Adult, Aged and LPS-Treated Rats

**Figure 7:**
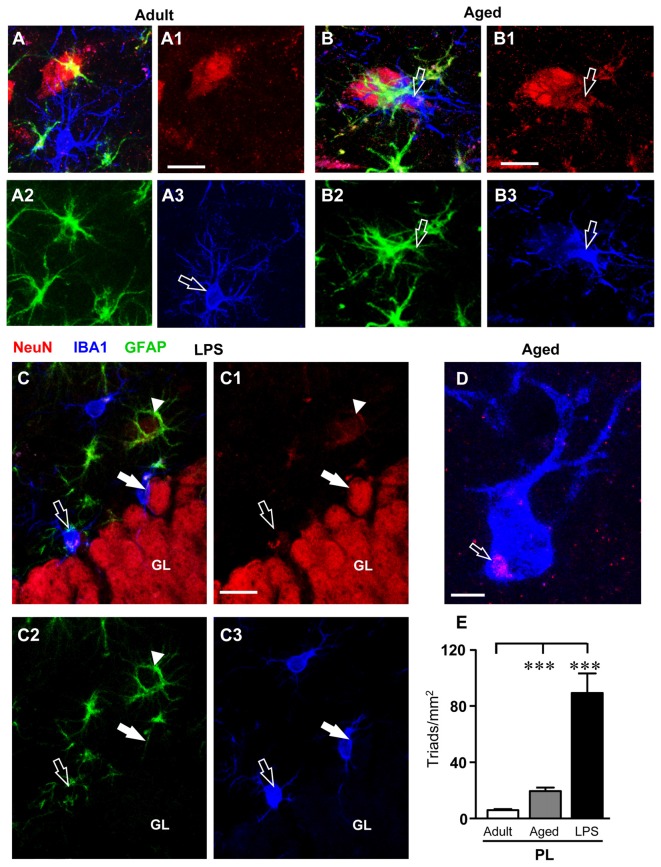
Quantification and characterization of the neuron-astrocyte-microglia triads in PL of adult, aged and LPS-treated rats. **(A–A3,B–B3,C–C3)** Confocal microscopy 3D renderings of triple immunostaining of neurons (NeuN, red), astrocytes (GFAP, green) and microglia (IBA1, blue) in the PL of an adult **(A–A3)**, an aged **(B–B3)**, and of an LPS-treated rat **(C–C3)**. **(A–A3)** The images show a neuron, astrocytes and microglia in the PL of an adult rat, not forming a triad. Scale bar: 10 μm. **(B–B3)** The arrows indicates neurons **(B1)** showing signs of degeneration with surrounding GFAP+ astrocytes **(B2)** and a microglial cell in reactive, phagocytic state **(B3)**, forming a triad **(A)**. Scale bar: 5 μm.** (C–C3)** The open arrow in **(C1)** indicates a neuron showing signs of degeneration with surrounding GFAP+ astrocytes and microglial cells in reactive, phagocytic state **(C3)** involved in the triad formation **(C)**. Scale bar: 15 μm. **(D)** Representative photomicrograph of an activated microglia cell (IBA1, blue) engulfing a neuronal debris (NeuN, red, open arrow) in PL of an aged rat. Scale bar: 2 μm. **(E)** Quantitative analysis of neuron-astrocyte-microglia triads/mm^2^ in DG PL of adult (*n* = 6), aged (*n* = 5) and LPS-treated rats (*n* = 4). Triads were significantly more numerous in PL of aged and LPS-treated rats. Data reported in all graph bars are expressed as mean ± SEM. Statistical analysis was performed using ANOVA and Newman-Keuls Multiple Comparison Test: ***P < 0.001 vs. adult rats (see text for details).

Triple immunostaining for neurons, GFAP+ astrocytes and microglia was performed in the DG of adult, aged and LPS-treated rats. Representative 3D renderings of triple immunostaining of astrocytes, neurons and microglia (Figure 7) with anti-NeuN antibody (red, Figures 7A1–C1), anti-GFAP antibody (green, Figures 7A2–C2), and with anti-IBA1 antibody for microglia (blue, Figures 7A3–C3) in the PL of an adult (Figures 7A–A3), an aged (Figures 7B–B3), and of an LPS-treated rats (Figures 7C–C3) clearly shows that many neuron-astrocytes-microglia triads were found in the PL of aged and of LPS-treated rats (Figures 7B,C, merge). The 3D rendering in Figure 7A (stack of 53 consecutive confocal z-scans, 0.3 μm each, total thickness 15.9 μm), shows that in the PL of an adult rat astrocytes and microglia surrounded a neuron but did not form a triad. The microglia cell had morphological characteristics of a resting microglia, with a small cell body and long, thin branches (Figure 7A3, open arrow). The 3D rendering in Figure 7B (stack of 53 consecutive confocal z-scans, 0.3 μm each, total thickness 15.9 μm) shows that in the PL of an aged rat a damaged neuron was surrounded by two different GFAP+ astrocytes that sent their branches to form a micro scar around the neuron. A microglial cell (Figure 7B3) with phenotypical characteristics of reactive microglia, such as an enlarged cell body and short cellular processes (Miller and Streit, 2007), was in close proximity to the damaged neuron and was phagocytosing the cytoplasm, as shown by the pink color inside the microglia cytoplasm (Figure 7B, open arrow). The 3D rendering in Figure 7C (stack of 14 consecutive confocal z-scans, 0.3 μm each, total thickness 4.2 μm), shows that two damaged neurons, very close to the GL, formed triads with astrocytes and activated microglia cells which were engulfing the damaged neurons (Figure 7C, open arrow and white arrow). It is evident that both granular neurons were close but slightly detached from the GL. The open arrow indicates a neuron that has almost completely been phagocytized by the microglia cell, while the white arrow indicates a neuron that is starting to be attacked by the microglia cell. The arrowhead in Figure 7C shows an astrocyte forming a microscar around a degenerating neuron. Figure 7D (stack of six consecutive confocal z-scans, 0.3 μm each, total thickness 1.8 μm), taken in the PL of an aged rat shows the magnification of a digital subslicing (starting at about 4 μm inside the cell) of an amoeboid-shaped activated microglia that is phagocytosing a neuron (pink color, open arrow).

Quantitative analysis of neuron-astrocytes-microglia triads in the PL of adult, aged and LPS-treated rats showed that the triads increased by 170% in aged rats (***P < 0.001 aged vs. adult rats, *F*_(2,14)_ = 43.37), and by 887% in LPS-treated rats (***P < 0.001 LPS vs. adult rats, *F*_(2,14)_ = 43.37) in comparison to adult rats (Figure 7E).

### Increased Fractalkin (CX3CL1) Expression in DG of Adult, Aged and LPS-Treated Rats

**Figure 8:**
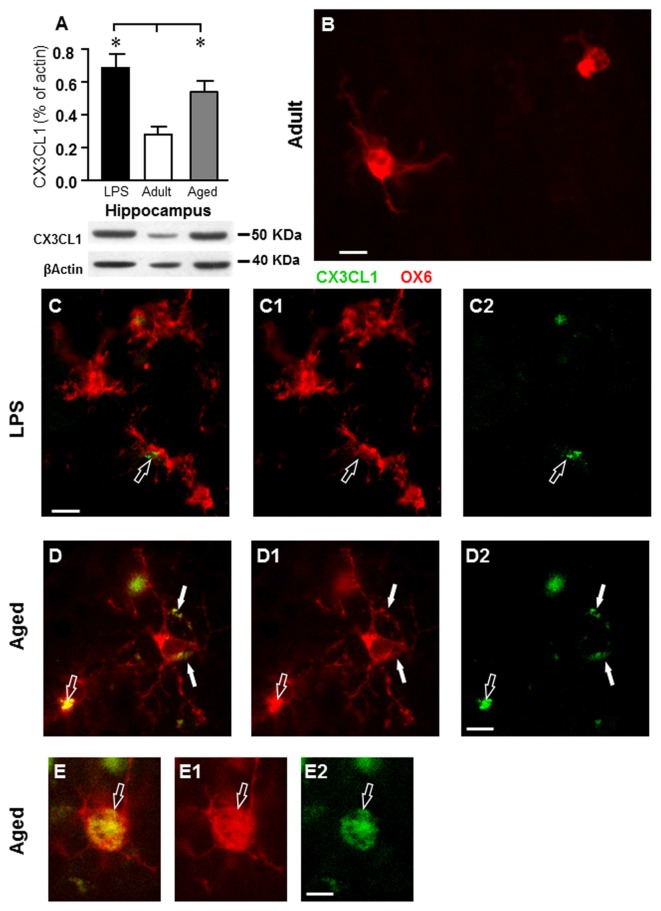
Analysis of CX3CL1 expression in the hippocampus of adult, aged and LPS-treated rats. **(A)** Quantitative Western Blot analysis of CX3CL1 in whole hippocampus homogenates of adult (*n* = 6), aged (*n* = 4), and LPS-treated (*n* = 4) rats. Each column in the graph represents the level of CX3CL1 normalized to β-actin run in the same gel, expressed as mean ± SEM (*P < 0.05 vs. adult rats). Typical Western Blots of CX3CL1 and actin run in the same gel are shown below. **(B–D2)** Fluorescent immunohistochemistry of CX3CL1 (**C2–D2**, green), of OX6 positive microglia (**C1–D1**, red), and the merge of CX3CL1 and OX6 **(B–D)** in the PL of an adult **(B)**, an LPS-treated rat **(C)** and of an aged rat **(D)**. **(B)** Scale bar: 5 μm; **(C–C2,D–D2)** Scale bar: 10 μm. These images show that CX3CL1 colocalized with microglia cells (arrows) in aged and LPS-treated rats. **(E–E2)** Representative photomicrographs demonstrating that CX3CL1 (**E2**, green) is expressed in the cytoplasm of an activated microglial cell (**E1**, OX6, red) in the PL of an aged rat. **(E)** Is the merge of the two previous images. Scale bar: 5 μm.

In accordance with previous data (Cerbai et al., 2012) quantitative WB analysis of CX3CL1 in homogenates of whole hippocampus of adult, aged rats and rats treated with LPS demonstrated that levels of CX3CL1 were significantly higher in aged (+80%), and in LPS-treated rats (+90) hippocampus than in adult rat hippocampus (*F*_(2,12)_ = 5.365; *P* < 0.005; **P < 0.05 vs. adult rats, Figure 8A). Double labeling immunofluorescent analysis of CX3CL1 (Figures 8C2–E2, green) and activated microglia (Figures 8B,C1–E1, red) showed that immunostaining of CX3CL1 colocalized in the cell body (open arrows) and in the branches (arrows) of activated microglia cells in the PL of aged and LPS-treated rats (Figures 8C–E), but not of adult rats (Figure 8B). Colocalization of CX3CL1 with neurons or astrocytes was never found in the DG of any of the three experimental groups (data not shown).

## Discussion

Communication among neurons and astrocytes-microglia is of particular interest in physiological and pathological conditions and can provide insights into the aging process and help identify biomarkers of aging. Here we studied the changes in the intercommunication among neurons, microglia and astrocytes in the DG of the hippocampus during aging and in response to acute experimental neuroinflammation induced by treatment with LPS. Therefore, we studied two different conditions, one characterized by chronic low-grade inflammation caused by aging and the other one by a more intense, subchronic inflammatory response caused by LPS. We focussed on the DG of the hippocampus as it represents the first link of the canonical trisynaptic pathway that conveys electrophysiological inputs from the enthorinal cortex to the hippocampus proper (Amaral, 1993; Amaral and Lavenex, 2007; Witter, 2007). Particularly, our study was directed to understand the modifications that might occur in the GL and PL of the DG.

The progressive modifications that occur in the aging brain, or “inflammaging”, (Franceschi et al., 2007; Deleidi et al., 2015), are characterized by chronic, low-grade, upregulation of several pro-inflammatory mechanisms and by changes in the reciprocal intercellular communication in the triads among neurons, astrocytes and microglia (Cerbai et al., 2012; Lana et al., 2014, 2016, 2017) that cause neuroinflammation. Here we demonstrated that in the GL and PL of aged and LPS-treated rats astrocytes were less numerous than in adult rats. Nevertheless, in the GL of LPS-treated rats the GFAP+ astrocytes acquired the morphology of reactive astrocytes, with principal branches longer than astrocytes of adult rats. Total and activated microglia increased in aged rats and in rats treated with LPS. Mainly in the GL of aged but also, to a lesser extent, in the GL of LPS-treated rats many neurons showed signs of apoptosis. Consistent with these results, the number of granular neurons decreased significantly in GL and PL of aged rats. This effect was not evident in GL and PL of LPS-treated rats, suggesting that the subchronic neuroinflammation was insufficient to reproduce a similar degree of granular cell loss. We found that in PL of aged and LPS-treated rats many damaged neurons were embraced by microglia and were infiltrated by astrocyte branches, which appeared to be bisecting the neuron to form cellular debris which were phagocytosed by reactive microglia. Triads were significantly more numerous in PL of aged and LPS-treated rats. This effect was consistent with microglia scavenging dying neurons. The levels of the chemokine CX3CL1 increased, and in the PL of aged and LPS-treated rats CX3CL1 immunoreactivity was colocalized both in the branches and in the cell body of activated microglia.

The networks of communication among different cells change during aging or disease, and this aspect is particularly true and can have great consequences in the brain. It is not clear whether age-related changes of intercommunication and interplay among different cell types are simply adaptations to aging, or actively contribute to aging or disease mechanisms *per se*. As a consequence, the interplay among different cell types may modulate or even control aging or may be unbalanced in particular diseases (De Keyser et al., 2008; Sofroniew, 2009). For a long time neurons have been considered the basic functional units of the central nervous system, and glia only trophic and supportive elements. However, recently it is becoming evident that for the functional organization of the brain proper intercommunication among cells that form the neuron-astrocyte-microglia “triad” is fundamental (Barres, 2008; Allen and Barres, 2009). We and others (Cerbai et al., 2012; Re et al., 2014; Lana et al., 2016) demonstrated that in stress conditions, astrocytes fragment degenerating neurons and cooperate with microglia in the disposal of neuronal debris.

In line with our previous data (Cerbai et al., 2012; Lana et al., 2016), here we found that many neurons in the GL of aged rat hippocampus underwent apoptosis, which caused cellular degeneration and death. The decrease of neurons in DG of aged rats, possibly made more significant by reduction of neurogenesis during aging (Kuhn et al., 1996), may contribute to age-related memory impairments, as demonstrated in previous experiments with similar rat models (Lana et al., 2016). In the aged rat DG, not only neurons showed signs of degeneration, but astrocytes were less numerous and had morphological features of clasmatodendrosis (Hulse et al., 2004; Mercatelli et al., 2016). In a less neuron-centric view of neurodegeneration during aging, the loss of astrocytes and their functions such as brain homeostasis maintenance, extracellular glutamate and ion buffering, as well as energy and nutrient supply to neurons, may contribute to spread of neural damage and degeneration (Miller et al., 2017). It has been demonstrated (Bernal and Peterson, 2011) that the decrease of astrocytes in DG of aged rats is accompanied by decreased astrocyte-dependent VEGF expression during aging, further supporting our findings. Nevertheless, the findings in this regards are still controversial (for review see Rodríguez-Arellano et al., 2016).

The current investigation did not find significant decrease of neurons in DG of LPS-treated rats. Since LPS is detrimental for neurogenesis (Ekdahl et al., 2003; Littlefield et al., 2015), other mechanisms must be taken into consideration to explain this apparent discrepancy. First, apoptotic neurons in GL of LPS-treated rats, although more numerous than in adult rats, were significantly less numerous than in aged rats, and the consequent the neuronal death may be less relevant. Furthermore, although in LPS-treated rats, as in aged rats, astrocytes were less numerous than in adult rats, in LPS-treated rats astrocytes were in a reactive state. Indeed, astrocyte branches were longer, and were able to pass through the entire depth of the GL, a finding indicative of a better trophic support exerted by astrocytes towards granular cells in LPS-treated rats. This phenomenon, contrary to that observed in CA1 (Lana et al., 2014), can be considered a protective effect of astrocytes towards neurons.

Taken together with our previous reports (Cerbai et al., 2012; Lana et al., 2014, 2016), our findings confirm that reactive astrogliosis is not a single, uniform process and not always a negative phenomenon. In moderate astrogliosis, astrocytes have hypertrophic bodies and processes (Wilhelmsson et al., 2006), are distributed in contiguous, non-overlapping domains (Bushong et al., 2002), their proliferation is limited and do not form scars. In line with these findings, it has it been shown that adaptive astrogliosis is beneficial for neurons, while suppression of astroglia reactivity may increase neuronal vulnerability, exacerbating the pathological progression and altering regeneration (Sofroniew, 2009; Burda and Sofroniew, 2014; Pekny et al., 2014). Supporting our findings, other data demonstrated that hypertrophy of astrocytes may reflect astrocytes adaptive plasticity, as demonstrated in aged rodents increasing morphological complexity by an enriched environment (Rodríguez et al., 2013; Sampedro-Piquero et al., 2014).

Here we showed that many neurons that form triads with astrocytes and microglia in the PL of the DG were granular cells, located very close to the GL, although clearly detached from it. These results are in agreement with the current knowledge that during the first steps of apoptosis caspases break the cell cytoskeleton, allowing the apoptotic cell to detach from the surrounding, healthy cells (Böhm, 2003). This mechanism may explain how apoptotic, damaged neurons migrate from the GL to the PL to form triads in which phagocytosis may take place. Active and controlled cell death may serve a homeostatic function in regulating the number of cell population in healthy and pathological conditions (Kerr et al., 1972; Becker and Bonni, 2004). Thus, triad formation seems a specific mechanism for disposal of degenerating neurons, not only through phagocytosis, but also through the mechanism of phagoptosis (Brown and Neher, 2012). Phagoptosis is triggered by cell stress which is too mild to cause cell death, too serious to allow adaptation of the neuron to the damage but sufficient to recruit astrocytes and microglia for phagocytosis (Kao et al., 2011).

Microglia activation has been long considered detrimental for neuron survival, more recently it appears that this is not always the case (Solito and Sastre, 2012; Zhu et al., 2016). Furthermore, given the increased number of total and activated microglia cells in the DG of rats treated with LPS, we can hypothesize that the scavenging processes were more effective in DG of LPS-treated rats than in aged rats. These data are in agreement with results that showed that during aging, although microglia increased, the cells had morphological modifications that caused less neuroprotective and defensive capabilities of microglia (Streit et al., 2009; Tremblay et al., 2012; Streit and Xue, 2013).

In the current study, we confirmed that hippocampal levels of CX3CL1 (Cerbai et al., 2012) increased significantly both in aged and LPS-treated rats. At the morphological level, we found that CX3CL1 was never colocalized with neurons or astrocytes, but only with activated microglia. This is an interesting, unexpected finding since, as reported by Luo et al. (2016), although CX3CL1 is considered to be principally expressed by neurons, while its receptor by microglia (Harrison et al., 1998; Cardona et al., 2006; Lauro et al., 2008), it is still debatable whether other cell types also express CX3CL1. We had previously demonstrated that CX3CL1 immunostaining in CA1 was localized on neurons phagocytized by microglia (Cerbai et al., 2012). Nevertheless, in the periphery CX3CL1 is expressed in different inflammatory conditions by monocytes, macrophages and other cells types such as fibroblasts, endothelial cells, and dendritic cells (Jones et al., 2010). Therefore, as the resident macrophage cells of the brain, it is possible that in particular areas and in certain stress conditions such as inflammation, microglia may express CX3CL1. Consistent with these data we also found a highly significant increase not only of total but also of activated microglia in PL of LPS-treated rats. On the other hand, microglia express the only receptor for CX3CL1, whose role in the CX3CL1-associated activation of microglia is well known (Jung et al., 2000). Therefore, it is also plausible that immunofluorescence of CX3CL1 that we detected on microglia might depend upon the binding of CX3CL1 to its receptor. Indeed, although it has been shown that CX3CL1 maintains microglia in a quiescent state (Lyons et al., 2009; Bachstetter et al., 2011), it has also been demonstrated that soluble CX3CL1 increases and is released in cerebral ischemia (Dénes et al., 2008), in response to apoptosis (Fuller and Van Eldik, 2008) and to glutamate excitotoxicity (Chapman et al., 2000) but its role as a neuroprotective or neurotoxic molecule remains unresolved (Lauro et al., 2015). It has been shown that CX3CL1 is neuroprotective in cultured rat hippocampal neurons (Limatola et al., 2005; Cipriani et al., 2011) and *Cx3cr1*^−/−^ mice show reduced damage after cerebral ischemia; this protection may be due to the anti-inflammatory state of local microglia (Tang et al., 2014). CX3CL1 may also be or deleterious (Liu et al., 2015) in different models of neurodegenerative diseases, indicating that the effects of CX3CL1 may be different, according upon different degenerative stimuli (Lauro et al., 2008).

### Comparison of the Results Obtained in Studies of the DG, CA1 and CA3

It is generally believed that neuroinflammation is characterized by astroglia activation which can be typified by morphological changes, accompanied by low to moderate levels of inflammatory mediators in the parenchyma. Although it is commonly agreed that astroglia is activated and reacts similarly in different conditions (Ransohoff, 2016) and brain areas, our data demonstrate the responses of astrocytes and microglia to aging and LPS-induced inflammation to the same stressful stimuli are different not only among different subregions but also within the same hippocampal subregion. The differential reactivity of astrocytes and microglia is reported in Table 2, which is built from results taken from our present data and from previous published articles (Cerbai et al., 2012; Lana et al., 2016), all obtained in the same rat models of aging and brain inflammation. From the data reported in Table 2 it is interesting to note that in all hippocampal subregions of aged rats, astrocytes decreased significantly, while total microglia decreased in CA1 only, and increased in CA3 and DG. In LPS-treated rats both total and activated microglia increased in all three regions, while astrocytes did not vary in CA1 Stratum Radiatum (SR), increased in CA3 SR and decreased in DG PL. Of note is also the much lower density of activated microglia in CA1 in comparison to CA3 and DG, in the three experimental models. Thus, taken together with the results from our previous investigations of the hippocampus under identical conditions, we conclude that in DG PL and in CA1 and CA3 SR (Cerbai et al., 2012; Lana et al., 2016), all subregions of rat hippocampus that are contiguous and interconnected, astrocytes and microglia show very different reactivity in the three experimental groups. These data demonstrate that astrocytes and microglial responses to the same insult are not uniform, but vary significantly from area to area and in different stress conditions. It will be of great interest to confirm whether these differences of glial reactivity may explain the differential susceptibility of the hippocampal areas to aging or to different inflammatory insults (Masgrau et al., 2017).

**Table 2:** Density of GFAP+ astrocytes, resting and activated microglia in stratum radiatum (SR) of CA1 and CA3 and polymorphic layer (PL) of dentate gyrus (DG) of adult, aged and LPS-treated rats.

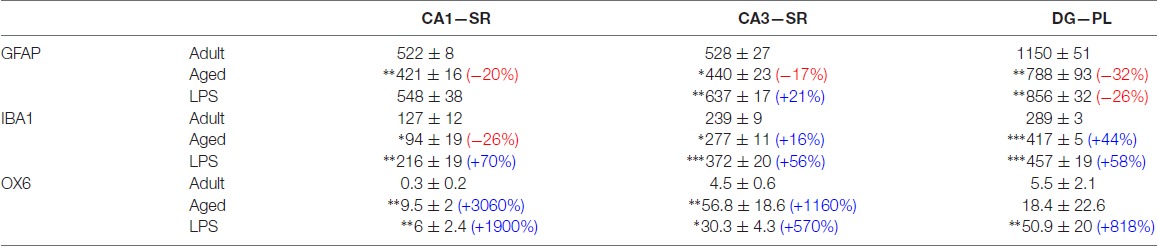

Significant increases, expressed as percent of adult rats, are shown in blue, significant decreases in red. CA1—SR: from Cerbai et al. (2012); CA3—SR: from Lana et al. (2016). All statistical analyses were performed using ANOVA and Newman-Keuls Multiple Comparison Test: *P < 0.05 vs. adult rats, **P < 0.01 vs. adult rats, ***P < 0.001 vs. adult rats (see text for details).

## Conclusion

In conclusion, here we show that in the DG of aged and LPS-treated rats, astrocytes and microglia participate in phagocytosis/phagoptosis of apoptotic granular neurons. The differential expression/activation of astrocytes and microglia in CA1, CA3, DG and the alteration of their intercommunication may be responsible for the differential susceptibility of the three hippocampal areas to neurodegeneration during aging and inflammation.

## Author Contributions

MGG, DL and FU designed the research; DL, FU and DN performed the experiments; DL, FU and MGG analyzed the data; DL, MGG and GLW interpreted the results and the experiments; DL, MGG and FU prepared the figures, MGG drafted the manuscript; DL, MGG, FU and GLW edited and revised the manuscript; DL, FU and MGG read and approved the final version of the manuscript.

## Conflict of Interest Statement

The authors declare that the research was conducted in the absence of any commercial or financial relationships that could be construed as a potential conflict of interest.

## Footnotes

^1^http://rsb.info.nih.gov/ij
